# Vertically sheathing laminar flow-based immunoassay using simultaneous diffusion-driven immune reactions†

**DOI:** 10.1039/c9ra03855h

**Published:** 2019-07-31

**Authors:** Amanzhol Kurmashev, Seyong Kwon, Je-Kyun Park, Joo H. Kang

**Affiliations:** Department of Biomedical Engineering, School of Life Sciences, Ulsan National Institute of Science and Technology (UNIST) Ulsan Republic of Korea jookang@unist.ac.kr +82-52-217-2639 +82-52-217-2595; Department of Bio and Brain Engineering, Korea Advanced Institute of Science and Technology (KAIST) Daejeon Republic of Korea

## Abstract

We present that enhanced simultaneous incubation of multiple antibodies (Abs) can be achieved by exploiting microfluidic laminar flows and difference in diffusivity between primary Ab (pAb) and secondary Ab (sAb). We demonstrate that injecting Ab of larger and smaller diffusivity (*D*_Ab_) in an upper and lower laminar flow over the analyte-coated bottom surface, respectively, would result in enhanced signal intensity in the given reaction time. To prove this, we simultaneously infused anti-prostate specific antigen (PSA) pAb (upper laminar flow) and quantum dot (QD) labeled secondary Ab (QD-sAb) (lower laminar flow) to generate two Ab laminar flows vertically sheathing each other in the microfluidic device in which PSA was immobilized on the glass bottom surface. Because of the larger *D*_Ab_ of pAb than that of QD-sAb due to the heavy metal components of QD, anti-PSA pAb diffuses more rapidly toward the bottom surface where the immune reaction between PSA, pAb, and QD-sAb instantaneously occurs. We corroborated our principle by switching the position of the two Ab laminar flows (QD-sAb in upper and pAb in lower laminar flows) in the channel, which resulted in significantly lower intensity of QD signals than the previous method. Moreover, when we adjusted the interface of pAb and QD-sAb in upper and lower laminar flows, respectively, closer toward the bottom surface, the fluorescence signal was even more intensified. This is attributed to the increased flux of anti-PSA pAb more adjacent to the reaction site, which, in turn, enhances the binding efficiency of pAb to PSA on the surface.

## Abstract 2

Simultaneous infusion of primary and secondary antibodies of different diffusivity into vertical laminar flows enables the improved immune reactions.

## Graphical abstract

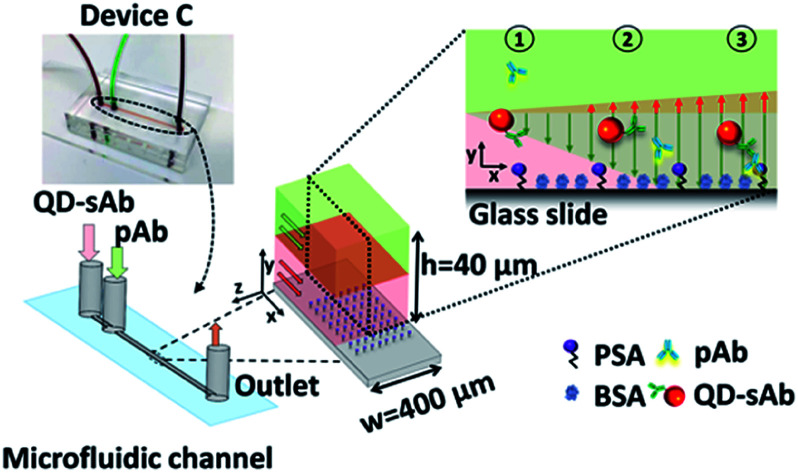

## Introduction

Conventional immunoassay methods, including enzyme-linked immunosorbent assays (ELISA), have become primary tools for the biochemical analysis of clinically important biomolecules, such as biomarkers for various diseases, hormones, viruses, and bacteria due to their high sensitivity and specificity.^1,2^ However, despite its wide use as a gold standard for a modern immunological assay platform, the overall ELISA process remains time-consuming and labour-intensive, which often requires multiple serial mixing and washing steps.^3^ These drawbacks of the conventional immunoassay platforms are attributed to the prolonged incubation steps, which are due to the inefficient mass transport imposed by slow and infrequent delivery of antibody molecules to analytes immobilized on the solid matrix surface.^4,5^ Leveraging microfluidic principles for delivering reagents to the immunoreaction sites has alleviated this issue by considerably reducing the diffusion distances and allowing constant replenishment of the antibody molecules at the reaction site.^6–8^ The additional utility of the microfluidic immunoassays was to reduce the reagent volumes consumed, to incorporate multiple processes in a single device, and to significantly lower overall analysis time.^9,10^

To quantify the presence of the target molecules in liquid samples, the conventional microfluidic devices often deliver antibody molecules in a stepwise-flowing manner for a multi-step immunoassay.^11,12^ For fluorescence immunoassay, the intensity of the detection signals emitted in this case not only indicates the rate of immunoreaction that occurs between the analyte and antibody molecules but also highly correlates with the rate of mass transport of the antibody molecules from bulk solutions to the reaction site.^13^ In fact, for the vast majority of the known immunoassay mechanisms, immunoreactions between antigens and antibodies occur “instantaneously”, which implies that the rate-limiting step of the immunoassay is the diffusion of antibody molecules to the reaction site.^14^

Various microfluidics-based immunoassay methods have been developed to address the limitations of conventional assay platforms. They exploited a variety of different approaches, such as the automated lab-on-a-disc devices,^15,16^ integration of an optical system in the microfluidic devices,^17–19^ a quantum dot (QD) “barcoding” system,^20,21^ and the three-dimensional microfluidic confinement.^22,23^ Despite considerable efforts in the microfluidic immunoassay platforms in the past decades, most researches have focused on automation,^24–26^ increased surface areas,^27,28^ improved reporter probes,^29,30^ and distinctive detection principles;^31,32^ however, the efficient delivery of antibody reagents to the reaction sites has been rarely explored.

In this work, we report that a significantly improved immune reaction can be achieved by using simultaneous infusion of primary and secondary antibodies by exploiting the difference in diffusivity between antibody reagents flowing in the vertically sheathing laminar streams in a microfluidic channel. We also demonstrate the more preferable experimental conditions when primary antibody (pAb) and secondary antibody (sAb) are simultaneously infused into a microfluidic channel where analytes are immobilized.

## Experimental

### Reagents and instrumentation

Natural human prostate-specific antigen (PSA) protein (ab78528) and rabbit polyclonal anti-PSA antibody (anti-PSA pAb) (ab9537) were purchased from Abcam (Cambridge, MA, USA). Bovine serum albumin (BSA) was purchased from Sigma-Aldrich (St. Louis, MO, USA). Qdot 625 modified F(ab′)2-goat anti-rabbit IgG (H + L) sAb (A-10194) were obtained from Thermo Fisher Scientific (Waltham, MA, USA). All solutions were prepared using 1× phosphate-buffered saline (PBS) buffer purchased from Biosesang (Sungnam, Korea). Glass slides coated with aldehyde groups were purchased from LumiNano (Seoul, Korea). Precision syringe pumps (CHEMYX, Fusion 200, TX, USA), 1 mL gas-tight syringes (Hamilton Company, NV, USA), 23G 0.5′′ blunt needles (SAI Infusion Technologies, IL, USA) and Tygon® microbore tubing (ID = 0.02 IN, Saint-Gobain Korea, Seoul, Korea) were used to control flows of liquids and injection of samples into microfluidic channels.

All computational simulations were completed with the commercially available finite element method software, COMSOL® (COMSOL, Inc., Burlington, MA, USA). The parameters provided to the numerical analysis included diffusion coefficients of antibodies (*D*_1_ = 6.3 × 10^−11^ m^2^ s^−1^ for anti-PSA pAb; *D*_2_ = 2.4 × 10^−11^ m^2^ s^−1^ for QD-sAb), their concentrations (20 nM for QD-sAb; 30 nM for anti-PSA pAb), dimensions of the microfluidic channel (width = 40 μm, length = 15 mm), and the flow rates at which the solutions were infused (50 μL h^−1^ for both anti-PSA pAb and QD-sAb solutions). The diffusion coefficients for anti-PSA pAb and QD-sAb were estimated using the Stokes–Einstein equation,^33^ based on molecular weight (150 kDa) and hydrodynamic radius (10 nm), respectively.

### Fabrication of the microfluidic device and reversible bonding strategy

**Fig. 1:**
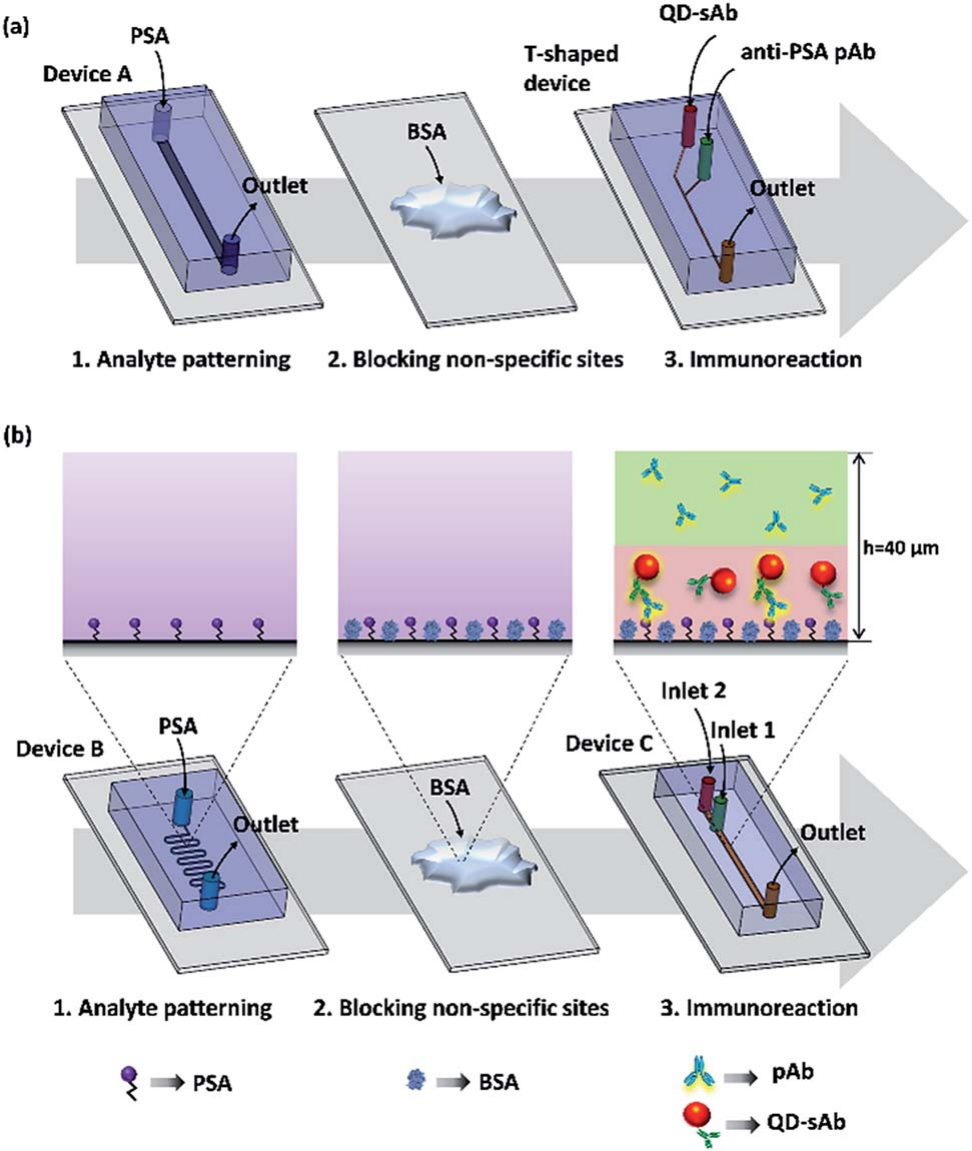
A schematic representation of the experimental protocol. (a) Lateral diffusion-based immunoassay in a microfluidic T-shaped device. (b) Vertically sheathing laminar flow-based immunoassay in the microfluidic Device C.

Polydimethylsiloxane (PDMS) devices were fabricated by a standard soft lithography process using a Sylgard 184 silicone elastomer kit purchased from Dow-Corning (Midland, MI, USA) and a mold fabricated with SU-8 negative photoresist (MicroChem Corp., Newton, MA, USA). For an immunoassay in a T-shaped device, the analyte (PSA) was first immobilized on an aldehyde glass slide by infusion of the PSA solution (20 μg mL^−1^) at a flow rate of 10 μL h^−1^ through a straight channel using the Device A (1 mm × 40 μm × 15 mm; width × height × length) (Fig. 1a). The T-shaped device also contains a straight channel with a rectangular cross-section (40 μm × 40 μm × 15 mm; width × height × length) that has two inlets and one outlet ports (Fig. 1a). To demonstrate the principle of the immunoassay we proposed, we immobilized the analyte (PSA) on the aldehyde-coated glass slide substrate using the Device B of the serpentine microfluidic channel (100 μm × 40 μm × 212 mm; width × height × length) (Fig. 1b). We then simultaneously injected two antibody reagents solutions (pAb and sAb) using the Device C that contains a straight rectangular channel (400 μm × 40 μm × 37 mm; width × height × length) with two inlets and a single outlet. A metallic pressing device (0.35 kg) was placed onto the PDMS device to ensure secure reversible sealing between the PDMS channel and the glass slide surface (Fig. S1†). The design of the pressing device and its operation principle were adopted from the previous research.^34^

### Immunoassay procedures

Lateral diffusion-based immunoassay experiment for evaluating and visualizing the difference in diffusion rates of the antibodies was carried out by infusing the solutions of anti-PSA pAb (30 nM) and QD-sAb (20 nM) through the channel of the microfluidic T-shaped device that is placed over the PSA-patterned area on the aldehyde glass slide. Both solutions were infused at a flow rate of 50 μL h^−1^ for 20 min. Since the channel of the T-shaped device (width = 40 μm) is significantly narrower than the channel of Device A (width = 1 mm), it can be easily aligned with the PSA-patterned area without employing the serpentine-shaped channel (Device B).

Vertically sheathing laminar flow-based immunoassay that we propose in this work begins with flowing the PSA solution (20 μg mL^−1^) at a flow rate of 10 μL h^−1^ for 2 h through a serpentine microfluidic channel of the Device B (Fig. 1b) to immobilize PSA on the glass slide. By using a serpentine channel, we were able to immobilize PSA on a wide area of the aldehyde glass slide surface, which facilitated subsequent alignment of the microfluidic channel of Device C for injecting primary and secondary antibody solutions. We then removed the PDMS device and thoroughly washed the analyte-patterned area of the substrate using 1× PBS. After this, non-specific binding sites on the PSA patterns were quenched with 3% BSA for 15 min. After another PBS washing, the microfluidic Device C (Fig. 1b) with two inlets and one outlet was placed onto the target area (PSA patterned) and was reversibly sealed using the pressing device to infuse the pAb and QD-sAb solutions for subsequent immunoreactions. To implement the vertically sheathing laminar flow-based immunoassay, anti-PSA pAb solution (30 nM) and QD-sAb solution (20 nM) were infused into the inlet 1 and inlet 2 of the Device C, respectively, at a flow rate of 50 μL h^−1^ for 20 min. At low Reynolds number (0.14), the pAb and QD-sAb solutions in an upper and lower laminar stream, respectively, are flowing through the microfluidic channel without convective mixing (the experimental condition 1). To validate our proposed principle, we switched the inlets of the pAb and QD-sAb solutions (the experimental condition 2) and infused QD-sAb and pAb solutions in an upper and lower laminar flow, respectively, in the channel. In each experiment, about 17 μL of each antibody solution (pAb and QD-sAb) was consumed for one round of immunoassay. Furthermore, to position the interface of pAb and QD-sAb solutions closer to the bottom surface of Device C, we changed the flow rates to 20 μL h^−1^ and 80 μL h^−1^ for QD-sAb (lower laminar flow) and pAb (upper laminar flow), respectively. For comparison, we also flowed the solution of pAb and QD-sAb solutions, mixed at a ratio of 1 : 1 and 1 : 4, at a flow rate of 100 μL h^−1^ for 20 min (the experimental condition 3). The amount of the antibody reagents (pAb and QD-sAb) for each experiment was consistently retained throughout all experiments.

We measured the fluorescence signals of QD-sAb immobilized on the glass slides after removing the PDMS devices from the glass slide to completely exclude the signals that could have been emitted from QD-sAb non-specifically bound to the surface of the PDMS channel. A fluorescent microscope (CKX53; Olympus, Japan) and a long pass filter were used to assess fluorescent signals emitted from Qdot 625 conjugated with the secondary Ab. All images were captured using a CMOS camera (DigiRetina 16, Tucsen, China) and analyzed using ImageJ (NIH, USA).

Significant differences in all experimental data were determined by the ANOVA test, as defined *P* values < 0.05. The results were presented as an average of at least triplicate samples ± standard error of the mean depicted by error bars in all graphs.

## Results and discussion

### Computational simulation of interdiffusion of antibody reagents in a microfluidic channel

**Fig. 2:**
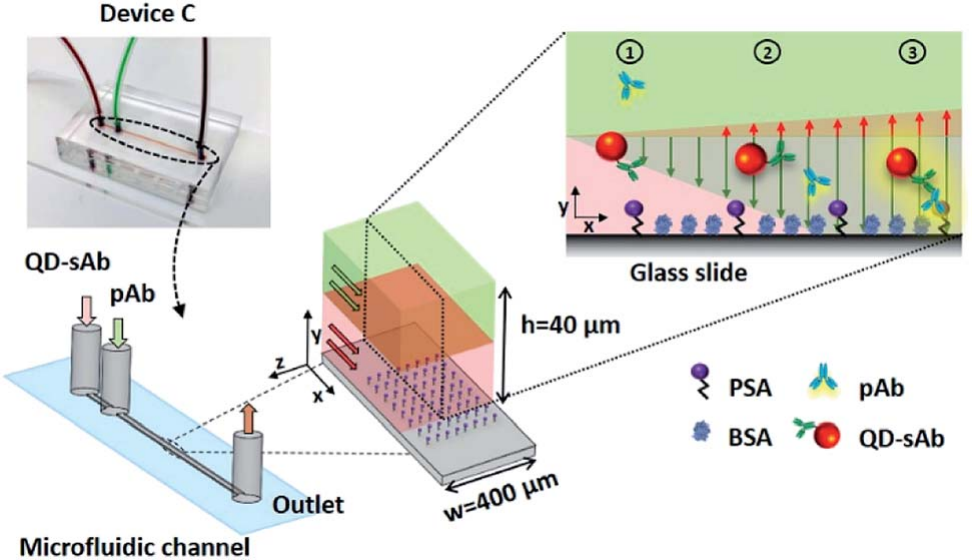
The principle of the proposed microfluidic immunoassay based on vertically sheathing laminar flows of pAb and QD-sAb using Device C. When pAb and QD-sAb are flowing in the upper and lower laminar flows, respectively, pAb diffuses more rapidly to the reaction site where the immunoreactions between PSA, pAb, and QD-sAb instantaneously occur.

We predicted that the immunofluorescence signals on the bottom surface would be significantly enhanced when simultaneously injecting pAb (*D*_1_ = 6.3 × 10^−11^ m^2^ s^−1^) in the upper laminar flow and QD-sAb (*D*_2_ = 2.4 × 10^−11^ m^2^ s^−1^) in the lower laminar flow because pAbs diffuse more rapidly toward the reaction site on the bottom surface and achieve instantaneous immune reactions of PSA, anti-PSA pAb, and QD-sAb (Fig. 2). We set out to validate our prediction by switching the position of the pAb and QD-sAb in the vertical laminar flows, which was predicted to result in much-reduced signal intensity because QD-sAb with *D*_2_ smaller than *D*_1_ more slowly diffuses toward the bottom surface while flowing in the laminar flows. Moreover, the fluorescence signals are predicted to be even more augmented when we adjust the vertical position of the laminar flow interface between pAb and QD-sAb closer toward the bottom surface where the PSA molecules are immobilized. This is attributed to that the utmost flux of pAb is formed in the interface between two laminar flows because the flux is proportional to the concentration gradients.

To validate our theoretical prediction, we carried out computational simulation using COMSOL Multiphysics software. We simulated the two laminar flows flowing through the T-shaped microfluidic channel with two inlets and one outlet to visualize the molecular mass transport across the channel in a lateral direction. The width of the T-shaped channel corresponds to the height of the channel in Device C, and this T-shaped model allows us to simply visualize and quantitate the diffusion between the laminar flows, which occurs in Device C in a vertical direction. Thus, the concentration profiles of antibodies across the width of the T-shaped microchannel approximate their actual diffusion in a vertical direction toward the reaction site on the bottom surface in Device C. The interpretation of our proposed diffusion in the T-shaped device is valid because the mass transport is mainly determined by diffusion, not by the gravitational force, in this microscale regime, and thus, the axis along which the diffusion takes places can be assumed negligible.

**Fig. 3:**
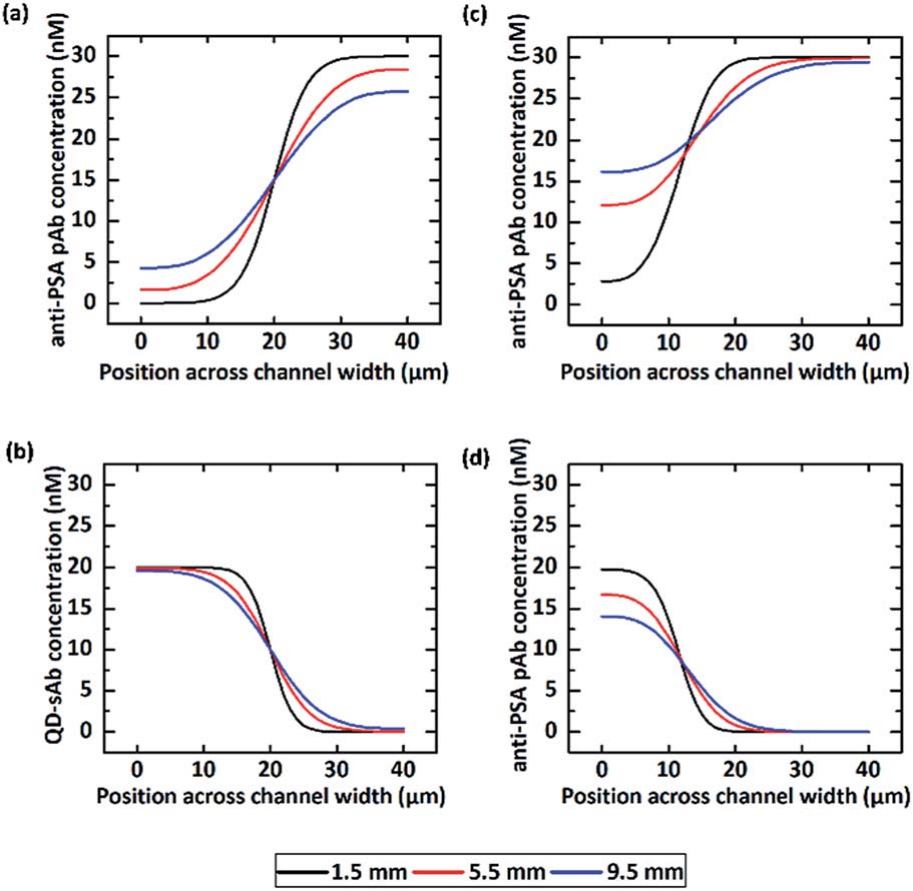
The concentration profiles across the T-shaped microfluidic channel at each longitudinal position were predicted by COMSOL®. When pAb and QD-sAb were infused into the device at a flow rate ratio of 1 : 1, we obtained the concentration profiles of (a) anti-PSA pAb and (b) QD-sAb. The concentration profiles were also predicted when (c) anti-PSA pAb and (d) QD-sAb were injected at a ratio of 4 : 1. The interface between two reagent solutions was shifted toward the right side of the channel due to the asymmetric input flow rates.

We first calculated the concentration profile of anti-PSA pAb and QD-sAb solutions when they were injected into the T-channel device at a flow rate of 50 μL h^−1^ (the flow rate ratio = 1 : 1). The simulation results (Fig. 3a) indicate that the initial concentration profile of anti-PSA pAb at 1.5 mm downstream from the inlet undergoes considerable changes at 5.5 mm and 9.5 mm due to rapid diffusion of pAb molecules across the channel while that of QD-sAb presents insignificant changes at 5.5 mm and 9.5 mm along the microfluidic channel due to their relatively larger molecular weight (Fig. 3b). If we change the ratio of the flow rate between the QD-sAb and pAb solutions from 1 : 1 to 1 : 4, the interface was shifted toward the left side of the channel and the change in the concentration profile between the two positions along the channel (1.5 mm and 5.5 mm) became even more significant (Fig. 3c and d), which is most likely due to the slower flow rate near the channel wall.^35^ In this case, because the concentration gradients of pAb became closer toward the surface of the right side in the T-channel device, which corresponds to the bottom surface of Device C, we predicted that the higher flux of pAb to the reaction sites could be achieved in Device C.

### Lateral diffusion-based immunoassays in a microfluidic T-shaped device

**Fig. 4:**
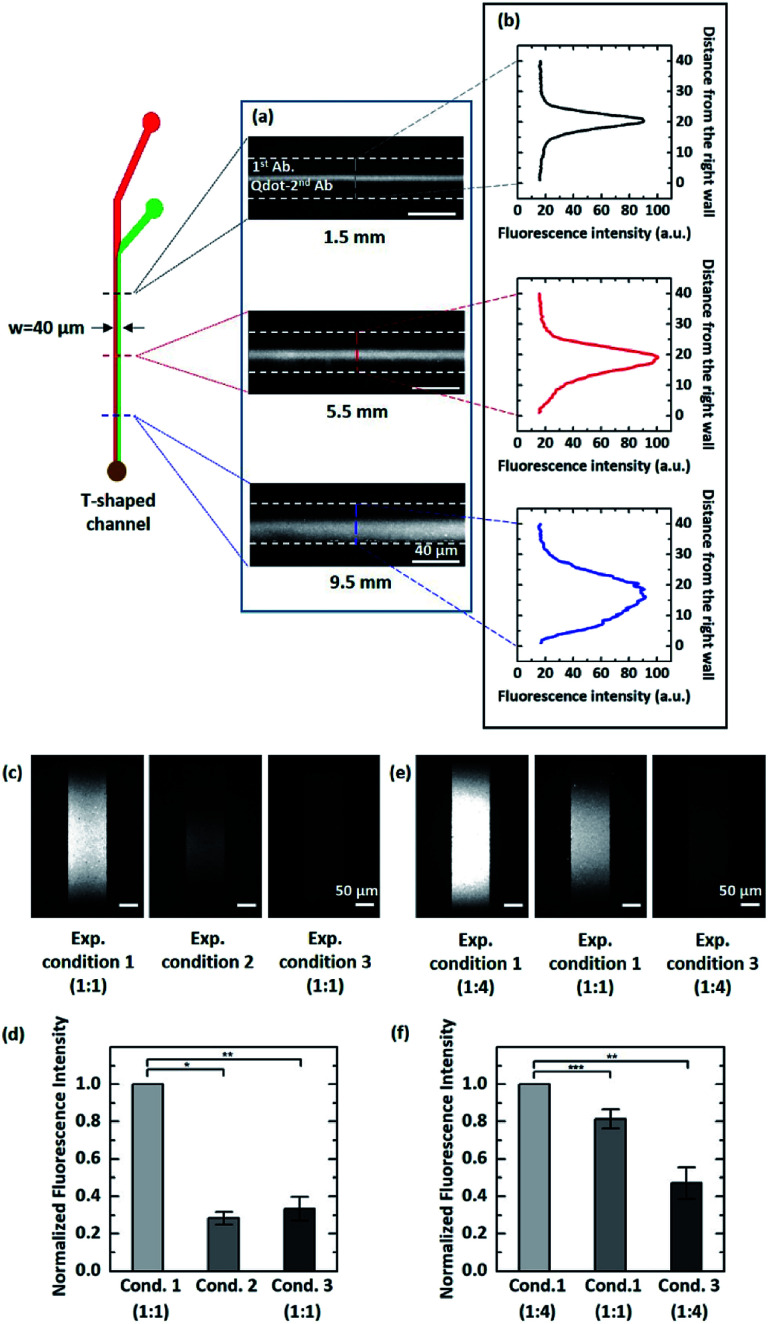
(a) The fluorescence microscopy images obtained at each longitudinal position along the channel of the T-shaped device. (b) The quantitative fluorescence signal intensity measured across the channel of the T-shaped device corresponding to each fluorescence image in (a). (c) and (e) The fluorescence microscopy images of the immune reaction zones on the glass substrates after immunoassay in the Device C. The distance between the inlet 1 of Device C and the position of the fluorescence measurement is 9.5 mm while in the exp. condition 1 (1 : 4), it is 3 mm. (d) and (f) The normalized signal intensities obtained from the corresponding methods of Abs infusion in (c) and (e). All data are expressed as the average ± S.E.M. from three independent experiments. The *P* value was calculated from the ANOVA test. *, *P* < 0.0005; **, *P* < 0.005; ***, *P* < 0.05.

To confirm that our theoretical prediction is valid, we carried out the experiment using pAb and QD-sAb solutions (the flow rate ratio = 1 : 1) in the T-shaped microfluidic device. Because PSA was immobilized on the bottom surface of the glass slides, we assessed the immunoreaction of pAb and QD-sAb on the PSA molecules by measuring the QD signal intensity emitted from QD-sAb (Fig. 4a and b). Although the fluorescence images obtained at each position (1.5 mm, 5.5 mm, and 9.5 mm) along the T-shaped channel did not reveal the real-time concentration profile of each reagent (pAb and QD-Ab), we indirectly assessed the relative interdiffusion of pAb and QD-sAb by assessing the fluorescence intensity, which is proportional to the reaction rate dominated by diffusion of each molecule to PSA on the surface. The fluorescence signals were formed symmetrically across the channel when the pAb and QD-sAb flowed for a relatively short distance from the inlet (1.5 mm and 5.5 mm) because the interdiffusion in a lateral direction across the channel between pAb and QD-sAb did not have enough time to result in significant differences of the fluxes of each reagent (Fig. 4b top and middle). However, as they flowed for a distance over 9.5 mm, we observed apparently asymmetric fluorescence signal distributions across the channel (Fig. 4b, bottom), which is attributed to the more rapid diffusion of pAb toward the right side of the channel in comparison to diffusion of QD-sAb toward the left side of the channel. With this experimental validation, we predicted that we could enhance the fluorescence signals if we inject antibody reagent solutions with larger *D*_pAb_ and smaller *D*_QD-sAb_ in the upper and lower laminar flow, respectively, in Device C.

### The simultaneous infusion of antibody reagents for immunoassay

We experimentally validated our proposed principle by infusing pAb (sheathing in the upper laminar flow) and QD-sAb over the PSA-immobilized surface in Device C. According to Fick's first law, the rate of immune reactions in mass-transport limited circumstances is determined by the flux of antibody reagents.^36^ In this case, delivering pAb to the reaction site is a rate-limiting factor because pAb needs to bind to the PSA molecules on the bottom surface prior to being recognized by QD-sAb. Thus, the concentration gradients of pAb proportionate to the pAb flux toward the surface are a determinant in the sequential immune reactions between PSA, pAb, and QD-sAb, and the fluorescence signals are predicted to be significantly enhanced as we position the interface of pAb and QD-sAb closer to the PSA-immobilized bottom surface. In contrast, if the QD-sAb and pAb solutions are injected in the upper and the lower laminar flows, respectively, into Device C, this would lead to a considerable reduction in the fluorescence signals because QD-sAb inefficiently diffuses to the bottom surface due to its relatively smaller diffusion coefficient (*D*_2_).

As we predicted, the significantly enhanced fluorescence signals were obtained in the experimental condition 1 in comparison to the experimental condition 2 (Fig. 4c and d), and it became even more intensified when we adjusted the position of the interface between pAb and QD-Ab closer toward the bottom surface (Fig. 4e and f). We normalized the fluorescence signal to the highest fluorescence signals (the experimental condition 1) as we compared the relative fluorescence intensity.

We also infused a pre-mixed solution of pAb and QD-sAb into Device C (the experimental condition 3), however the fluorescence signals were much lower (0.333 ± 0.063) than that we obtained with the experimental condition 1, which is due to the higher molecular weight of the pAb-QD sAb complexes compared to that of individual antibody molecules and the Fab domains of QD-sAb already occupied by pAb molecules while mixing.

## Conclusions

Most immunoassays involve pAb and reporter probe-conjugated sAb to quantitate the target molecules immobilized on a solid matrix. Because the reporter-probes often include catalytic enzymes, fluorescence dyes, and QDs, the diffusion coefficient of the sAb tends to be smaller than that of pAb. Thus, exploiting the difference of diffusivity of antibody reagents flowing in parallel in laminar flows, we can significantly improve the efficiency of immune reactions with pAb and sAb by simultaneously infusing those antibody solutions in a microfluidic channel. The pAb solution with larger *D*_pAb_ is more desirable to be injected in the upper laminar flow, sheathing sAb of smaller *D*_sAb_ in the lower laminar flow, to enhance the signals that are gained from the reporter probes conjugated with secondary Ab. This strategy allows us to exclude the washing steps in the immunoassay procedure, which are commonly required in the conventional microfluidic immunoassays. While pAb diffuses through the laminar flows to the reaction surface, the antigen-binding domains of pAb interact with QD-sAb, which could reduce the accessibility of the binding domain to PSA due to steric hindrance. However, this effect was not significant in our experiment due to the continuous replenishment of unbound pAbs from the upper laminar flow. Moreover, the signal intensity could be further improved when adjusting the interface of pAb and sAb closer to the surface where analytes are immobilized and consequent immunoreactions take place. For example, we could employ the additional third laminar flow on top of the two laminar flows of pAb and QD-sAb, which would push the interface between pAb and QD-sAb even closer to the substrate surface. This would enhance the mass transport of pAb toward the reaction surface, resulting in the improved rate of immune reactions in a given condition.

## Conflicts of interest

There are no conflicts to declare.

## Supplementary Material

RA-009-C9RA03855H-s001
