# Supplementary material for: Vertically sheathing laminar flow-based immunoassay using simultaneous diffusion-driven immune reactions
Source: RSC Adv. 2019 Jul 31;9(41):23791–6. doi: 10.1039/c9ra03855h (PMC9069447; doi:10.1039/c9ra03855h)
Supplement: RA-009-C9RA03855H-s001 [file RA-009-C9RA03855H-s001.pdf]

Electronic Supplementary Material (ESI) for RSC Advances.
This journal is © The Royal Society of Chemistry 2019

Supplementary Information

Vertically sheathing laminar flow-based immunoassay using simultaneous diffusion-driven immune reactions

Amanzhol Kurmashev [a, †] , Seyong Kwon[a, †], Je-Kyun Park[b] and Joo H. Kang[*a]

[a]Department of Biomedical Engineering, School of Life Sciences, Ulsan National Institute of Science and Technology (UNIST), Ulsan, Republic of Korea. Email: jookang@unist.ac.kr

[b]Department of Bio and Braining Engineering, Korea Advanced Institute of Science and Technology (KAIST), Daejeon, Republic of Korea.

SUPPLEMENTARY FIGURE LEGENDS

Fig. S1 A configuration of the pressing device used for reversible bonding. The upper plate combined with a microfluidic device is placed on the aldehyde glass slide, and a weight is mounted on the upper plate to create reversible sealing.

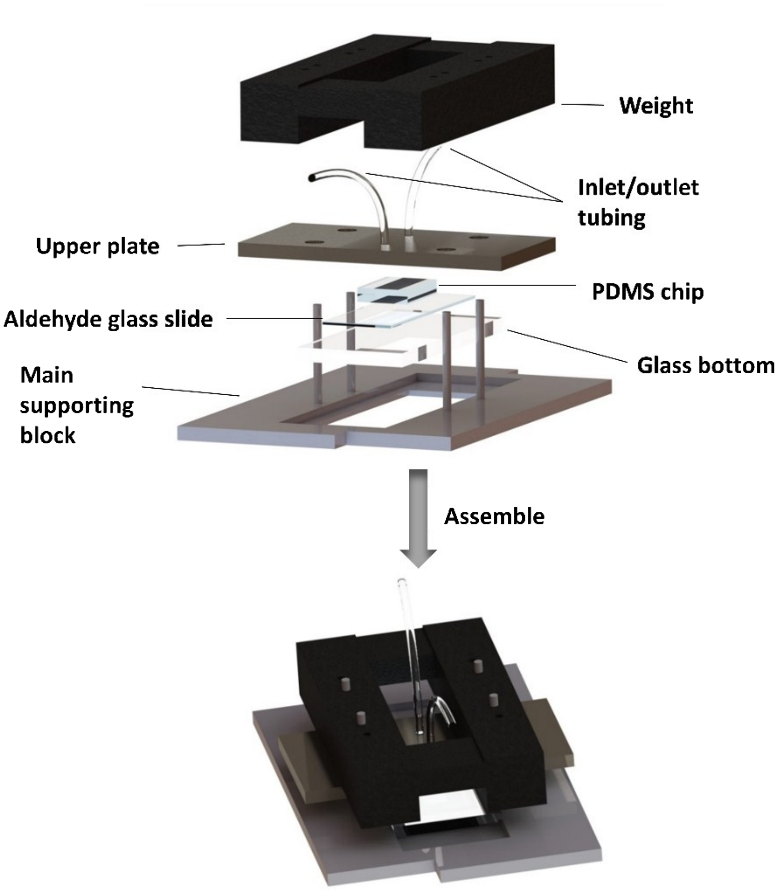

Weight
Inlet/outlet tubing
Upper plate
PDMS chip
Aldehyde glass slide
Glass bottom
Main supporting block
Assemble
